# ISBP: Understanding the Security Rule of Users' Information-Sharing Behaviors in Partnership

**DOI:** 10.1371/journal.pone.0151002

**Published:** 2016-03-07

**Authors:** Hongchen Wu, Xinjun Wang

**Affiliations:** School of Computer Science and Technology, Shandong University, Jinan, People’s Republic of China; Beihang University, CHINA

## Abstract

The rapid growth of social network data has given rise to high security awareness among users, especially when they exchange and share their personal information. However, because users have different feelings about sharing their information, they are often puzzled about who their partners for exchanging information can be and what information they can share. Is it possible to assist users in forming a partnership network in which they can exchange and share information with little worry? We propose a modified information sharing behavior prediction (ISBP) model that can help in understanding the underlying rules by which users share their information with partners in light of three common aspects: what types of items users are likely to share, what characteristics of users make them likely to share information, and what features of users’ sharing behavior are easy to predict. This model is applied with machine learning techniques in WEKA to predict users’ decisions pertaining to information sharing behavior and form them into trustable partnership networks by learning their features. In the experiment section, by using two real-life datasets consisting of citizens’ sharing behavior, we identify the effect of highly sensitive requests on sharing behavior adjacent to individual variables: the younger participants’ partners are more difficult to predict than those of the older participants, whereas the partners of people who are not computer majors are easier to predict than those of people who are computer majors. Based on these findings, we believe that it is necessary and feasible to offer users personalized suggestions on information sharing decisions, and this is pioneering work that could benefit college researchers focusing on user-centric strategies and website owners who want to collect more user information without raising their privacy awareness or losing their trustworthiness.

## Introduction

Taking one step beyond social network security, the fast growth of personal information sharing carries increasing risks and threats, and it is therefore not surprising that the privacy of information exchanges between users and their partners has attracted considerable attention from researchers, website owners, and users themselves [1, 2, 3]. Information disclosure is increasingly common on mobile social networks, including users’ location sharing [4–6], knowledge sharing [7–9], and identity sharing [10, 11]. Online social networks—e.g., Tencent WeChat, Facebook—and the associated sharing of content, comments, and experiences are widely utilized to facilitate relationships irrespective of physical distance [12, 13]. Users chat, talk, argue, comment, and bargain on these social networks to exchange contextual and local information [14]. However, information can be shared only for mutual benefit among users [15, 16]; for example: finding a restaurant for dinner on Google Maps means that a user must share his/her geographical location; looking for a pediatric surgeon requires the parent to disclose his/her child’s body information, such as gender and age; and acquiring auto care advice on a car repair network requires the car owner to input information on the car model, build year, odometer, and so on. As a result, we believe that information sharing-based network applications are essential for online users and website owners to overcome not only physical distances but also the trust gap between them [17], mainly relying on context-aware service [18]; however, this often may not be desirable for four reasons. First, exchanging contextual and demographic information may raise users’ privacy concerns. It is common for users to evaluate the benefit and risk when sharing information, which is called a “privacy calculus”. When a user thinks that there will be more benefits than risks, he/she is likely to share the information. Users’ evaluations of benefits and risks depend on their knowledge and experiences pertaining to the requested item and how much they trust the information recipient [19]. Second, it is common for users to share very little information with others, but they may be likely to disclose some information in exchange for discounts when checking out at the market or plaza. This interesting paradox has shown that users may lack certain background knowledge on managing what and with whom to share. Third, whether a user is willing to disclose information is individual-dependent and recipient-dependent. Some people may disclose nothing, whereas others would share everything. One user may share his credit card information with his family but be reluctant to disclose it to a stranger. Finally, users may have a cognitive effect, which means that they would “learn” and become more conservative. For example, at the beginning of the information collection process, users are likely to share low-sensitivity information, but they are reluctant to share other low-sensitivity requests after they refuse to disclose some highly sensitive information. This varied sharing behavior may indicate users’ cognitive effect, which could be further confirmed by machine learning techniques; e.g., the prediction accuracy is very low if users’ responses are varied or some users change their sharing patterns over time. To summarize, users’ privacy concern is one of the most important social issues connected to information technologies and users’ sharing behavior, and a personalized agent that can support users’ sharing decision making—e.g., helping them evaluate the consequent risk and benefit—is truly needed.

Many recent works have emphasized trust computing in social networks, showing that users cooperate and communicate with each other on the basis of trust and expand their partnership network by picking up other users they trust [20–22]. At the same time, other scholars emphasize the stability of social structure, in which users tend to trust their past information-exchange partners [23]. Further studies also showed that users’ psychological factors could help in predicting their sharing behavior, such as the trustworthiness, satisfaction, and usefulness of the requested item and information recipient [24]. Those studies are beneficial when the analysis starts with the users’ psychological features, but sometimes the data collected by researchers comprise mostly the feedback they request from the participants, while very little is known about the true characteristics of their users, especially their psychological feelings. This is mainly because users may lie to the questionnaires and hide their real information or simply try to please the questionnaire designer for higher payment. For example, an online questionnaire requires all users to fill their first internship experience and overall satisfaction. Some users simply click “good” on all items to obtain the payment faster; there is no way to distinguish whether they took the survey attentively, and their answers are mixed with the dataset consisting of hundreds and thousands of user samples. In contrast, our work avoids the noise as much as possible by not only setting up cheating tests but also, from the perspective of website designers, focusing on analyzing users’ demographic features—e.g., age, gender—which most users cannot hide and can be extracted from the social network more easily. The best advantage of analyzing users’ sharing behavior based on their demographic features is the simplicity of finding common criteria in the same cluster; for example, users have no difficulty understanding where they are from, their home address, etc., but may have different understanding of psychological features such as “very satisfied”, “satisfied”, “neutral”, “unsatisfied”, and “very unsatisfied”. The sparsity of data also means that collecting users’ emotional feature is more difficult than collecting demographic features; users require time to think about whether they are satisfied, whereas they can accept or reject sharing of their email addresses in no time.

Whereas many studies have been devoted to investigating various factors that drive information sharing, there is an interesting and unexplored tension in this body of work. Many aspects may influence one user’s decision regarding the sharing of his information, such as the type of the shared information, the user’s approach to evaluating the risks and benefits, and the demographic features of the user. Can we assist users by making the decision on which information can be shared and with whom they can share the information, for example, by predicting their preferences and future sharing decisions? If we can successfully predict one request that he will not share, we could skip that request and maintain this user’s satisfaction and trust, which may lead users to feel comfortable releasing and sharing more information. In this study, we propose an ISBP model that emphasizes users’ trust partnership formation and addresses the topic of predicting users’ information sharing behavior by exploring the factors that affect sharing behavior—e.g., gender, age, major, and the type of requested items. We test our hypotheses using data from two crowdsourcing datasets, and the experimental results provide some evidence that users’ sharing behavior is individual-dependent. This study is not only a pioneering work that applies ML to the dataset with information sharing behavior and a guideline for applying ML techniques in WEKA, but it also could benefit researchers and college staff who concentrate on user-centered strategy analysis and human–computer interactions in information sharing studies.

## Materials and Methods

### Ethic statement

The study was approved by the Ethics Committee of Research Center of Software and Data Engineering, Shandong University, China. Written informed consent was obtained by all the participants enrolled in this study.

### Hypothesis manipulation

In one of previous studies on users’ disclosure behavior to a recommendation system that handles client-side personalization [25], items were requested in an alternating fashion. Further analysis is made in the study of [26], which confirmed that the order of items requested would raise the variability and predictability of users’ disclosure pattern and lower the accuracy of prediction. Because the requested context info is generally more sensitive, this leads to requests of mixed sensitivity and also accentuates the uncommon context requests. In effect, we believe that users could be showing different sharing attitudes on demographic items (DI) and context items (CI):

H1a. The mean sharing amount shall be different between CI and demographic.

H1b. Users’ sharing behavior on DI should be more varied than on CI.

DI items are mainly about the information of users themselves as a natural or social individual, such as housing address, name, working title, etc. CI are mainly about the information users create when they browse the Internet, e.g., online purchases, IP addresses, email contents, etc. We create the first two hypotheses because we believe that users should be more familiar with their own demographic information than their contextual information. We will further classify the requested information into sensitive items (SI), mild items (MI), and non-sensitive items (NI) by ranking their sharing rate from users. Users’ features are the main factors we are going to explore with regard to their influence on users’ sharing behavior, so we conjecture:

H2a. Males should be more likely to share their personal information than females.

H2b. Males’ sharing behavior is less stable than females’.

The 2nd hypothesis is proposed based on females maybe being more cautious with their information sharing behavior such that they may be less likely to let others know exactly who they are and what they have. In addition, if they are withholding their information, their behavior will be less varied than males. Age should be considered similarly:

H3a. Younger participants are more likely to disclose their information than older participants.

H3b. Younger participants would show more varied behavior for information disclosures.

We propose the 3rd hypothesis because we believe younger people have gained less social experiences than older participants have. As a result, younger participants will consider fewer risks than older participants such that their information will be more readily disclosed. One explanation is that younger participants may have less information to disclose, e.g., disclosing a home address is more normal when a young user shares one apartment with other people, while older participants could prefer not to disclose their address due to their young children or grandchildren also living there. However, because younger participants may change their minds easily by performing the privacy calculus in the middle of the requests, they may exhibit different sharing decision making when faced with two equally sensitive requests: agree to disclose the information at the start of the questionnaire and then reject disclosing the information in the end. For example, a teenager will agree to connect his Facebook account to a game account for an additional game bonus, while a father who has two children will be less likely to share his online information no matter whether that request is made earlier or later.

H4a. Users who are performing computer-focused works or studies may disclose different amounts of personal information than other people.

H4b. Users who have majored in computer fields should show less varied sharing behavior than those who are not computer majors. We propose hypothesis 4 because we think users’ knowledge plays an essential role in evaluating risks and benefits, and that knowledge is closely related with people who have fruitful online experiences, such as computer engineers, website designer, etc. They would know that some items’ disclosures could cause serious consequences; therefore, they will show more stable sharing decisions. To confirm the result is not algorithm-dependent, we should try to learn the knowledge with different ML techniques. Some users may have varied sharing behavior such that capturing their sharing pattern would be difficult. We will first learn the knowledge and then pick up the prediction errors by combining different selections of factors, e.g., gender, age, and major.

### ISBP model base ML techniques

**Fig 1:**
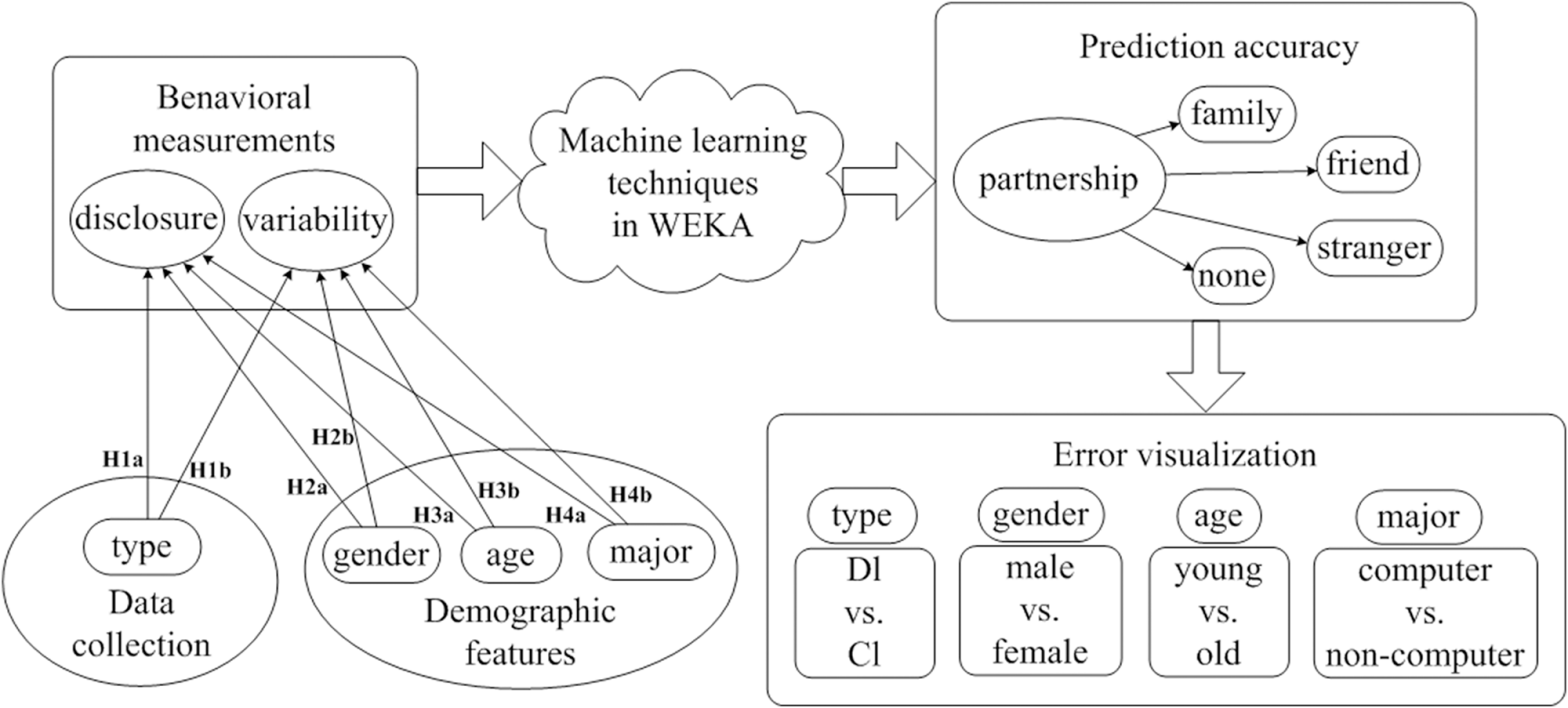
Workflow of the ISBP model.

We propose three ML techniques under the ISBP model shown in Fig 1, including how we load the data, how we train the knowledge, and what the results should look like. The knowledge that learnt was coming from users’ previous disclosing actions, and would be tested in the predictions of the following requested items. WEKA is an open-source and free software written in Java, developed at the University of Waikato, New Zealand (available at http://www.cs.waikato.ac.nz/ml/weka/). We use WEKA to implement our methods, and the ML techniques may obtain slight differences in predictions, but they should generate parallel results for testing the hypotheses.

As shown on the left side of Fig 1, we hypothesized that users’ features—e.g., gender (H2a & H2b), age (H3a & H3b), major (H4a & H4b), and type of requested items (H1a & H1b)—would affect their sharing behavior. We measure users’ sharing behavior by looking at their disclosure and variability. In the next step, we used ML methods, including decision tree, k-nearest neighbors, and naïve Bayes classifier, to predict the potential partners with whom users would place their trust and share their information, shown on the right side of Fig 1. Finally, the prediction errors were visualized according to 4 comparisons: demographic items vs. context items (type), male vs. female (gender), young vs. old (age), and computer vs. non-computer (major).

In the training set, all the users’ features and sharing behavior are included, e.g. their sharing decisions on previous requests with the recipients (family, friend, stranger, or none). ML techniques are applied to learn the underlying rules and predict further sharing action, together with prediction accuracy, recall and F-measure.

**Fig 2:**
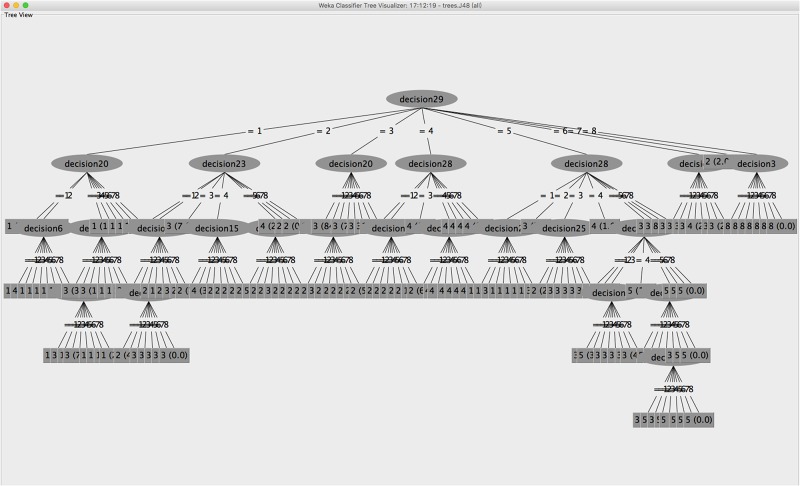
Visualized decision tree learning sample. The decision tree classifier will learn the regularity in users’ sharing behavior, and generate a flowchart-like structure starting from the root node, with paths connecting several leaf nodes and each representing a class label. As shown above, there are 57 users also agreed to share the item *20*, in the total of 63 users who had agreed to share the item *29*, so this rule is titled with 57/63 predicting accuracy.

Decision tree is the most widely applied supervised classification data mining technique, for it is simple and fast and can be applied in any domain [27]. A decision tree is a workflow-like structure that presents the logical connection between the values of attributes and the following outcomes with a class label. Any path from the top root to a leaf node stands for a classification rule, which is stored knowledge that could be further used for users’ sharing behavior predictions. A learnt tree can discover several trails starting from the root to many leaf nodes, split accordingly with one general sharing behavior of users to one requested item. Our ISBP model based decision tree classifier represents all the branches with several possible sharing action and their outcomes, and one of the visualized samples is shown in Fig 2.

The training set contains the records of users with known results, and this is used to generate the decision tree based on the sharing actions of the users and their various attributes, e.g., ID, gender, age, and majors, in response to 30 requested items with four options for the sharing candidates. The testing set is the unknown records of users, and this is used to test the decision tree developed from the training data. The fitness of each user’s real sharing actions and the predicted actions are compared.

The decision trees were generated by the C4.5 algorithm in WEKA from a set of training data using the concept of information entropy. The training set *S* = *s*_*1*_, *s*_*2*_…*s*_*n*_ is a set of classified users by an action to a certain item that we already know. Each user *s*_*i*_ consists of an *n*-dimensional vector (*x*_*1*,*i*_, *x*_*2*,*i*_,…,*x*_*n*,*i*_) that includes the attributes of users’ features (*x*_*1*,*i*_ to *x*_*j*,*i*_) and their sharing actions to items (*x*_*j+1*,*i*_ to *x*_*s*,*i*_). The last attribute *x*_*s*,*i*_ is the class in which *s*_*i*_ falls. C4.5 splits the set of users by picking the most effect way at each node on the way from the root to the leaf, enriching the subset in one class or the other, and a best splitting criterion is finding the highest information gain of the features. When a decision tree model is created, each user shall fall into a sublist of the data marked with his decision for sharing item *x*_*n*_. This model will be used on the testing dataset to see the matching percentage of the predicted decisions and their real decisions. The following is the pseudocode for the ISBP model-based decision tree generation.

Algorithm: ISBP model-based decision tree classifier

Input: Candidate users set *S* = (*s*_*1*_, *s*_*2*_, …,*s*_*n*_) and their action matrix. Each user *s*_*i*_ = (*x*_*1*,*i*_, *x*_*2*,*i*_,…,*x*_*n*,*i*_)

Output: Subsets of set *S*, and each subset represents a class in which some users fall.

Begin

Divide set *S* into 10-folds randomly, and each fold will be the testing set (*TE*) in turn; the rest of the folds are the training set (*TR*).

1. Root = DecisionTreeNode(*TR*)

2. dictionary = allUsers (*TR*, *x*_*n*_)

3. for routers in dictionary:

4.  if dictionary[router] = = total number of users

5.  node.label = router

6. return node

7. else if routers is empty

8.  node.label = the class where most users fit

9. return node

10. bestDecisionTree = the model with the highest information gain on attributes

11. set gain = entropy, sub = subset (*TR*, users with their features)

12. gain = gain—|sub|/(|*TR*| * entropy(sub))

13. if sub ≠ ∅

14.  *TR* = sub

15.   return to step 3

16.  else return bestDecisionTree

17. *x*_*i*_*’* = predict (bestDecisionTree, TE)

18. return RMSE, *p*-value, F-measure

End

The above code is for the prediction of users’ very last behavior *x*_*i*_ based on their features, such as age, gender, and major, and their previous actions. If we want to predict users’ actions on the behavior *x*_*i-1*_, the column *x*_*i*_ will be removed from the data, and the new data will be learned by the above code again. Running only one algorithm may not be persuasive enough to reach a good conclusion; therefore, we need to prove that any possible conclusion is not algorithm-dependent. The *k*-nearest neighbor classifier and naive Bayes Classifier are also applied in our dataset.

**Fig 3:**
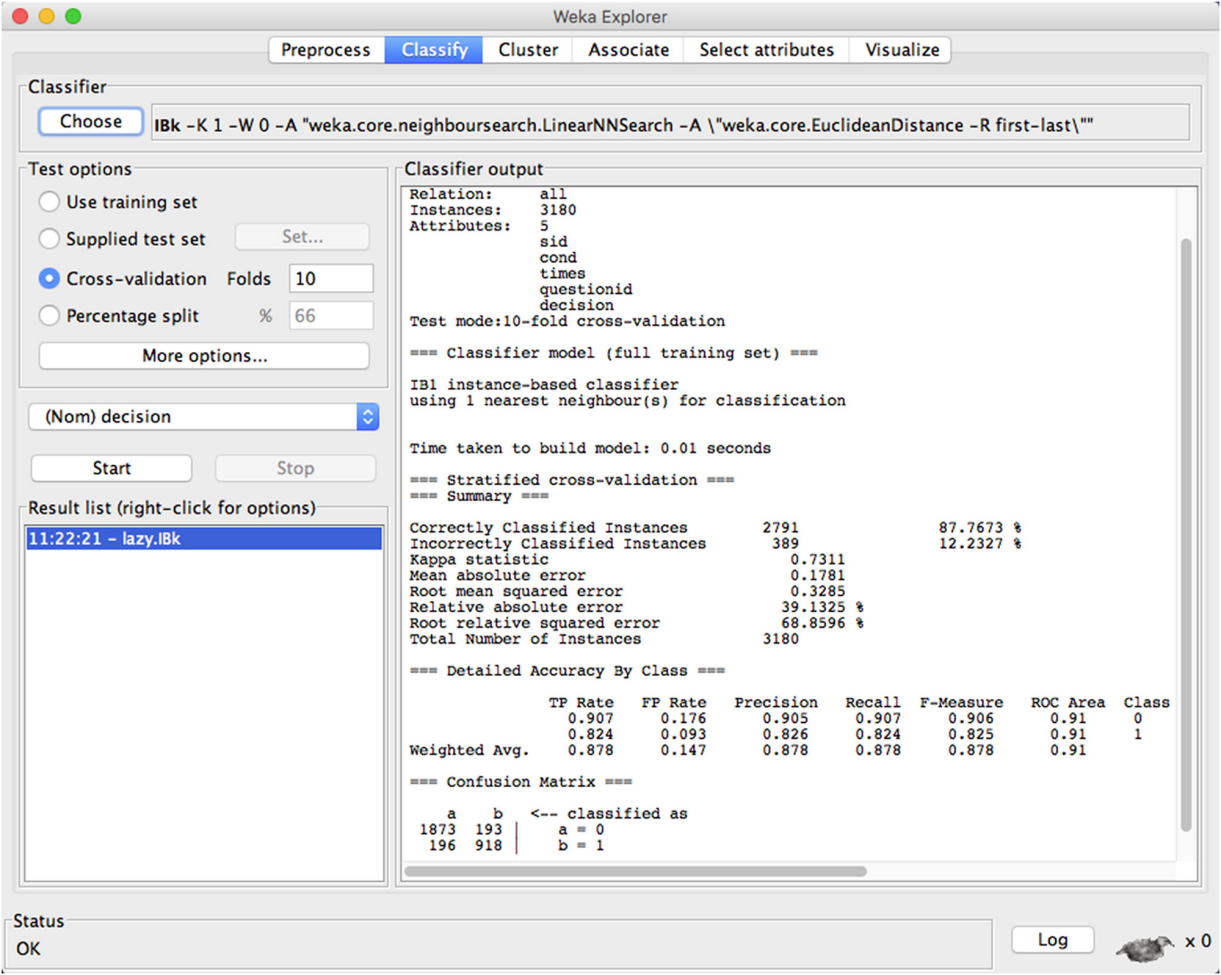
*K*-nearest neighbor sample. Suppose there is a dataset consisting a group of users’ sharing actions, WEKA will classify each user by a majority vote of its neighbors, assigning to the class most common among its nearest neighbors. The dataset will be randomly divided into 10 folds, and each fold will be used as the testing data while the rest will be applied as the training set. This sample regards *K* = 8, and the classified results indicate the precision is very high with over 87% percent of users are classified correctly.

The *k*-nearest neighbors algorithm is a non-parametric method of supervised learning for classification and regression. Unlike other ML techniques that require the explicit construction of featured spaces or high dimensions, the *k*-nearest neighbor classifier can be applied to learn the knowledge in a huge and highly varied dataset with less recognition efforts [28]. Our enhanced version of the ISBP based *k*-nearest neighbor classifier applied to predict users’ sharing behavior is: for a sample of user features and sharing actions *s*_*i*_, compute the distances of this sample to all other samples in the training set and find the nearest *K* neighbors; if most of the *K* neighbors are labeled with *x*, label *s*_*i*_ as *x* and exit. Fig 3 shows one sample that training users’ behavior with ISBP model based KNN classifier, and use the knowledge to predict their sharing actions to the 30th requested item, in which we receive high prediction accuracy (high precision, recall, and F-measures in predicting most classified results). Here is the pseudocode implemented for the prediction of the users’ sharing actions based on the *k*-nearest neighbor classifier.

Algorithm: ISBP model based *k*-nearcest neighbor classifier

Input: Candidate users set *S* = (*s*_*1*_, *s*_*2*_, …,*s*_*n*_), each user *s*_*i*_ is a vector of features and sharing actions {*x*_*i1*_, *x*_*i2*_,…,*x*_*in*_}.

Output: Subsets of set *S*, and each subset represents a class in which some users fall.

Begin

1. Let *R* = 0

2. for every instance *x*_*i*_ = *C* do score (*x*_*i*_) = 0

3. for every instance *y*_*i*_ = *T* do

4.  let *C*_*n*_ = 0

5.  add *y*_*i*_ *K* nearest neighbors in *C* to *C*_*n*_

6.  for every instance *x*_*j*_ = *C*_*n*_ do

7.   if *x*_*j*_ = *y*_*i*_ then score (*x*_*j*_) + = 1

8.   else score(*x*_*j*_) - = 1

9.  sort all instances by scores in descending order and the results to *R*

10. return *R*

After the model is built, each user in the training set belongs to a class, and for a user *y* in the testing set who is closest to most of users in a class *P*, give *y* as the same label.

**Fig 4:**
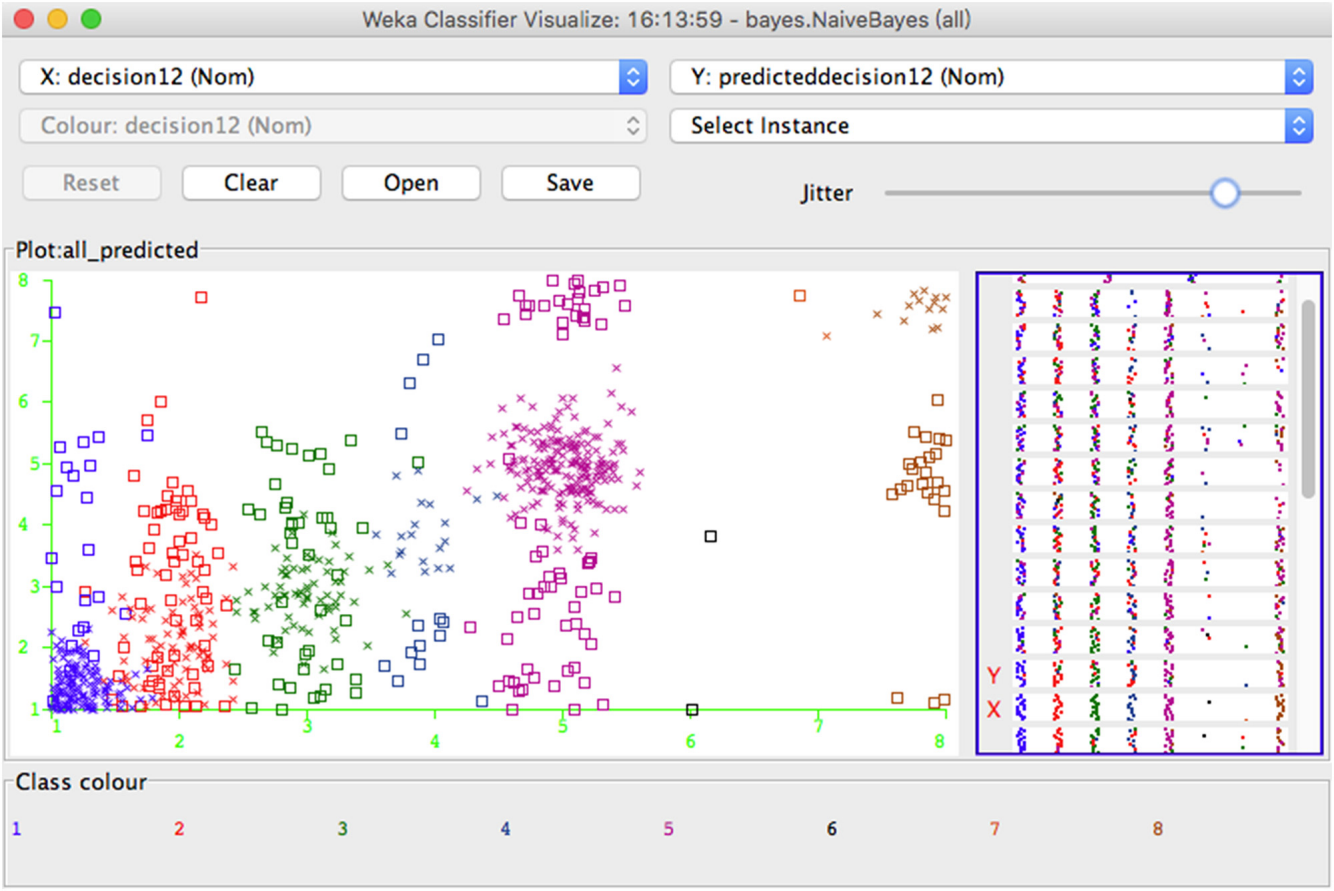
Naive Bayes Classifier Sample. The Naïve Bayes classifier will assign class labels to each user, represented as vectors of feature value, such as all the users who agreed to share item *1*–*7*, *13*–*17*, *24*–*29*, while refused to share item *8*–*12*, *18*–*23* will be classified into class *1*: “agree to share the item *12*”. This figure shows an example of Naïve Bayes with 8 identified classes.

The Naive Bayes classifier has been studied as a popular baseline method for categorization, and it is competitive with other advanced ML methods in all types of domains, such as automatic medical diagnosis [29], structured data such as atoms within molecules [30], etc. Naive Bayes classifiers can predict class membership probabilities, such as the probability that a given user sample belongs to a particular class, assuming class conditional independence. Although they can be applied in highly scalable data, e.g., users’ sharing behavior, they require a number of parameters linear to the number of variables in a learning model. The classified results of our ISBP based Naïve Bayes Classifier are shown in Fig 4, an information sharing behavior prediction model-based Naive Bayes classifier is: for a user *s*_*i*_ = {*x*_*i1*_, *x*_*i2*_,…*x*_*in*_} in the dataset *S* = {*s*_*1*_, *s*_*2*_,…, *s*_*n*_}, represent his features, such as age, gender, and major, and sharing behavior towards item *t*_*1*_, *t*_*2*_,…*t*_*m*_. Let *H* be the hypothesis such that sample *s*_*i*_ belongs to a specific class *C*, and, in our dataset, that is a hypothesis that this user *s*_*i*_ will share his/her information with the people *p*_*j*_ (family, friend, stranger, or none) on item *t*_*k*_. We need to determine P(*H*|*S*), which represents the probability that sample *S* belongs to class *C* and a posterior probability of hypothesis *H* conditioned on *S*, given that the attribute description of *S* is known. For example, a user *s*_*i*_ in *S* is a 40-year-old person who majored in computer science, and suppose that *H* is a hypothesis that he would be likely to share his current location information to a stranger, so P(*H*|*S*) is the probability that one user would share his/her location information with a stranger if we know his/her age and major. Furthermore, P(*H*) is the a priori probability of hypothesis *H*. In our dataset, it will be the probability that any user will disclose his/her location information to a stranger, regardless of the features of this user. In contrast, P(*S*|*H*) is the a priori probability of the hypothesis *H*. In our dataset, it will be the probability of a user who shares his location information with a stranger being 35-years-old and computer-majored. According to Bayes’ theorem, the probability P(*H*|*S*) is computed as:

$$P\left(H|S\right)=\frac{P(S|H)P\left(H\right)}{P\left(S\right)}$$ (1)

where P(*S*) is the percentage of users that are 35-year-old and majors in computer science.

The information sharing behavior partnership prediction model based naive Bayes classifier is as follows: Let *TR* be the training set of users, each with their class labeled as their sharing actions on item *t*_*x*_, where 7 classes in total, including *C1*: only share with family, *C2*: only share with friend, *C3*: only share with strangers, *C4*: share with family and friend, *C5*: share with family and stranger, *C6*: share with friend and stranger, and *C7*: share with nobody.

Each user *s*_*i*_ is represented by an *n*-dimensional vector *s*_*i*_ = {*x*_*i1*_,*x*_*i2*_,…*x*_*in*_} including his features of age, gender, major, etc., and sharing actions for the requested items. Given a user *s*_*i*_ in the testing set, the classifier will predict which class this user will belong to having the maximum expectation of a posteriori probability. That is, for any 1 ≤ *j* ≤ 7, user *s*_*i*_ will be predicted to belong to class *c*_*k*_, if and only if

$$P\left(C_{k}|s_{i}\right)>P(C_{j}|s_{i}),\text{ where }1\leq k\leq 7,\;k\neq j$$ (2)

Find the class *c*_*k*_ that maximizes P(*c*_*k*_|*s*_*i*_), which can be calculated in formula (1). Set all classes as equally likely, P(*C1*) = P(*C2*) = … = P(*C7*) = 1/7, at the beginning of the experiment because the a priori probabilities P(*Ci*) are unknown, and update their values as more users’ behavior are analyzed by P(*Ci*) = frequency (*Ci*,*TR*)/|*TR*|. To reduce the expense of computing P(*S*|*Ci*), we will assume that all features of users *x*_*ij*_ are independent of each other, and this will lead to:

$$P(s_{j}|C_{i})\approx \;\prod \begin{array}{l} n\\ k\;=\;1 \end{array}\;P(x_{jk}|C_{i})$$ (3)

and each probability P(*x*_*jk*_|*C*_*i*_) can be calculated as the frequency that any user’s feature *x*_*k*_ falls into class *C*_*i*_. P(*S*_*j*_|*C*_*i*_) is evaluated for each class *C*_*i*_ by predicting that the class label of *s*_*j*_ is *C*_*i*_ if and only if it is the class *C*_*i*_ that maximizes P(*S*_*j*_|*C*_*i*_)P(*C*_*i*_). To summarize, the pseudocode of the Naive Bayes classifier-based prediction of users’ sharing behavior is written as follows:

Algorithm: ISBP model-based Naive Bayes classifier

Input: seven classes for users, training set *TR*, testing set *TE*

Output: label each user in *TE* with one of the seven classes

Begin

1. while *TR* is not null

2.  calculate the distances among the users in *TR*

3.  initiate P(*C1*) = P(*C2*) = … = P(*C7*) = 1/7

4.  label every user *s*_*i*_ in TE with one class *C*_*k*_ (1 ≤ *k* ≤ 7)by highest expectation of *P*(*C*_*k*_ | *S*_*i*_)

5.  update *P*(*C*_*k*_) = ∂ × *P*(*C*_*k*_) + (1 − ∂) × |*P*(*C*_*k*_ | *S*_*i*_)|/|*P*(*S*_*i*_)|, *k* ∈ [1,7], ∂ ∈ (0, 1)

6.  {*C1*} + = *s*_*i*_, *TE* - = *S*_*i*_

7. return to step 1

END

## Results and Discussion

### Crowdsourcing platform and data preparation

Our data are collected from a crowdsourcing platform Sojump, which is a website providing online survey services that connects more than 2 million members throughout China and enables individuals and businesses to coordinate the use of human intelligence to perform tasks that computers are currently unable to complete [31, 32]. Using this online survey-based platform, the research collected data from nationwide users of social networks who joined our questionnaire globally. Each participant was required to give us his/her information on gender, age, and major before providing the 30 pieces of requested information. The survey was largely composed of three sections. The first section stated that the survey was conducted for academic research regarding online users’ sharing behavior and that no confidential information would be required from the participants. The second section required the participants to fill in their gender, age, and major. The last section consisted of 30 personal information requests, and participants were asked to consider which items they would agree to share with the following groups: family members, friends, strangers, or none. Multiple selections were allowed, but if a user chose the option “none,” we believe that this user rejects sharing of this item. We also set up a cheating test such that anyone choosing multiple selections including “none” would be excluded from further analysis.

**Fig 5:**
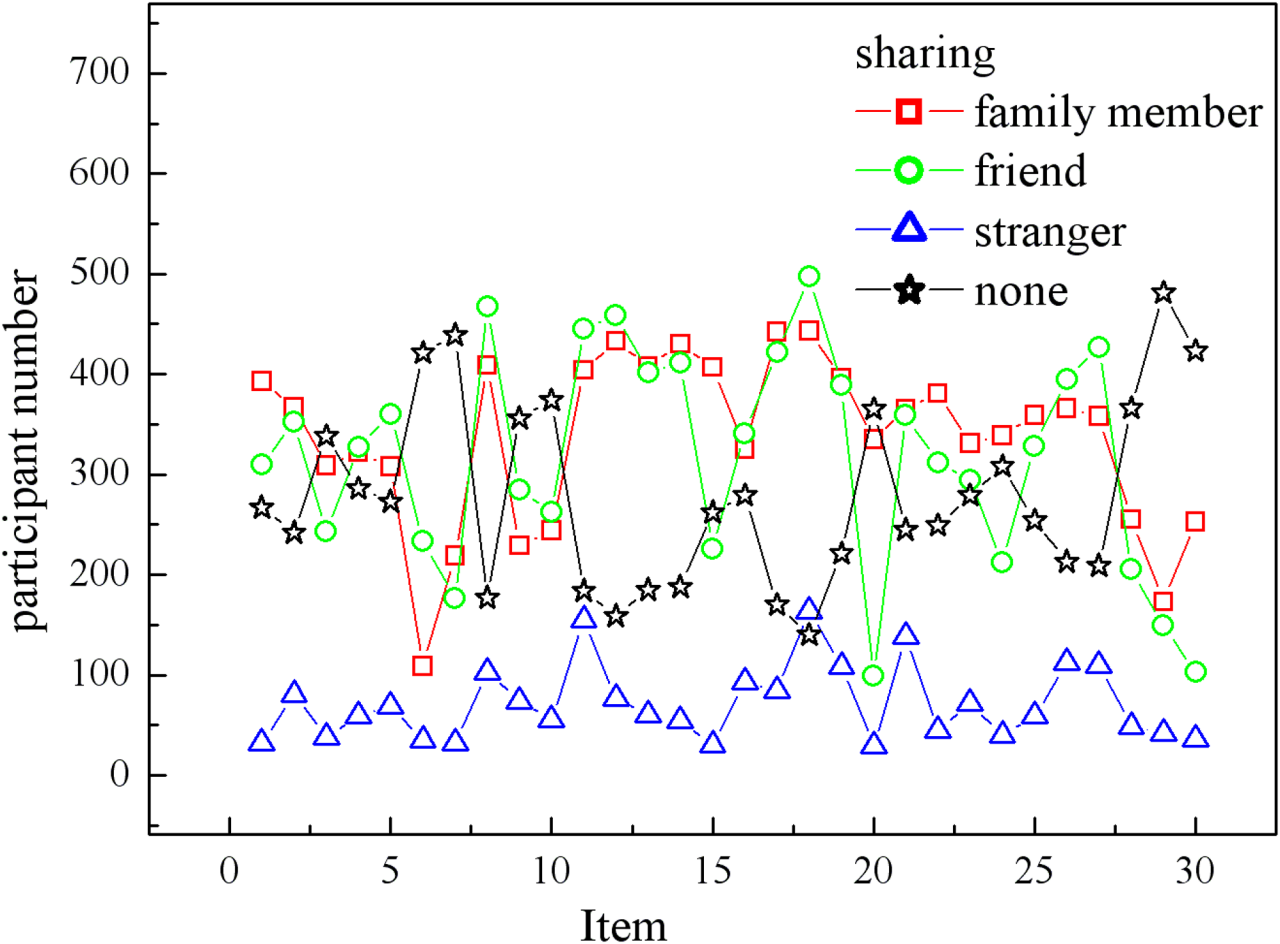
The sharing counts for each requested item with different recipients. Very few items were shared with strangers (triangles); more items were shared with friend (circles) and family (squares), and some items were preferred to share with nobody (stars).

The survey ran from March 20, 2015, to April 15, 2015; 860 participants from Sojump with unique IP addresses responded to our study, 774 of whom were qualified for further analysis, and the others did not pass the cheating test, see S1 File. The daily time spent on the Internet by each of the participants was more than 2 h, so we believe that the participants were all capable of basic knowledge with regard to privacy sharing. Sojump gave us a primary analysis of each requested item, and each participant should have been assigned different decisions for sharing depending on the people with whom this participant will share. Strangers, as we expected, received the lowest sharing points from the participants. However, to our surprise, friends received higher sharing points than family members. This is an interesting phenomenon in the experimental results, which are shown in Fig 5.

The 30 requested items are the most commonly requested information items in social networks, and they can be classified either by type (DI and CI) or by sensitiveness. Items can also be classified as SI (sensitive items), MI (mild items), and NI (non-sensitive items) by ranking their mean sharing rates. The 19 DI and 11 CI items all represent commonly requested information in social networks, and we tried to even up the number of SI, MI, and NI items by finding the personal information that users’ were most likely to disclose and least likely to disclose over the Internet.

We initially set three counters, $Fa_{SP}^{X}$, $\;Fr_{SP}^{X}$, and $St_{SP}^{X}$, to 0. If one user agrees to share an item *x* with his family members, *x*’s family sharing point $Fa_{SP}^{X}$ will be incremented by 1. If one user agrees to share an item *x* with his friends, *x*’s friends sharing point $\;Fr_{SP}^{X}$ will be incremented by 1. If one user agrees to share an item *x* with strangers, *x*’s stranger sharing point $St_{SP}^{X}$ will be incremented by 1. The values of these three counters will determine the general value $Item_{SP}^{X}$ for the item *x*. In a pilot study, we invited an additional 300 online users to answer the 30 requested items (they were not allowed to attend the main study), and the values of the three counters were 267: 161: 39 ≈ 7: 2: 1. As a result, we determine the sensitiveness of an item by adding all of its sharing points for all information recipients:

$$Item_{SP}^{X}=Fa_{SP}^{X}\;\times \;0.1\;+\;Fr_{SP}^{X}\;\times \;0.2\;+\;St_{SP}^{X}\times \;0.7$$ (4)

Finally, when we obtained the sharing points of all items, the items were ranked in ascending order, and the top 10 items were regarded as NI, the bottom 10 items were regarded as SI, and the remaining 10 items in the middle were regarded as MI. Table 1 provides the descriptive statistics for the 30 items as answered by the users in the main study.

**Table 1:** Descriptive statistics for the 30 items answered by the users.

Item	Mean	SD	$Item_{SP}^{X}$	Type	Sensitiveness
**Geographical location on phone app**	0.94	0.82	123.7	CI	MI
**Time shown on phone app**	1.03	0.89	163.8	CI	MI
**Dictaphone in your phone**	0.76	0.77	106.1	CI	SI
**Data plan of your phone**	0.91	0.86	138.9	CI	MI
**Downloaded phone app**	0.95	0.86	151.1	CI	MI
**Browsing history by phone**	0.59	0.73	82	CI	SI
**Calendar plan on your phone**	0.55	0.71	79.5	CI	SI
**Phone model**	1.29	0.95	206.4	CI	NI
**Daily data usage on your phone**	0.75	0.84	131.7	CI	MI
**Homepage of your phone browser**	0.72	0.82	115.3	CI	SI
**Race**	1.29	0.98	237.9	DI	NI
**Permanent address**	1.25	0.83	189	DI	NI
**Current location**	1.12	0.80	163.2	DI	MI
**Job and work**	1.15	0.83	163	DI	MI
**Monthly income**	0.85	0.72	106.9	DI	SI
**Daily time spent reading**	0.98	0.92	165.8	DI	NI
**Age**	1.22	0.86	187.4	DI	NI
**Highest education level**	1.42	0.95	258.5	DI	NI
**Religious belief**	1.15	0.95	193.7	DI	NI
**Bank balance**	0.59	0.62	73.6	DI	SI
**Population density**	1.11	0.98	205.6	DI	NI
**Daily routine to work**	0.95	0.79	132	DI	MI
**Daily time spent on TV**	0.90	0.84	142.3	DI	MI
**Child information**	0.76	0.72	104.3	DI	SI
**Social interactions**	0.96	0.82	142.8	DI	MI
**Favorite news topic**	1.13	0.90	194	DI	NI
**Favorite sports**	1.16	0.92	198.2	DI	NI
**Online orders**	0.66	0.71	100.8	CI	SI
**Email address**	0.47	0.67	76.5	CI	SI
**Bank loans**	0.51	0.61	71.1	DI	SI

### Hypothesis test for mean disclosures and standard deviation

**Fig 6:**
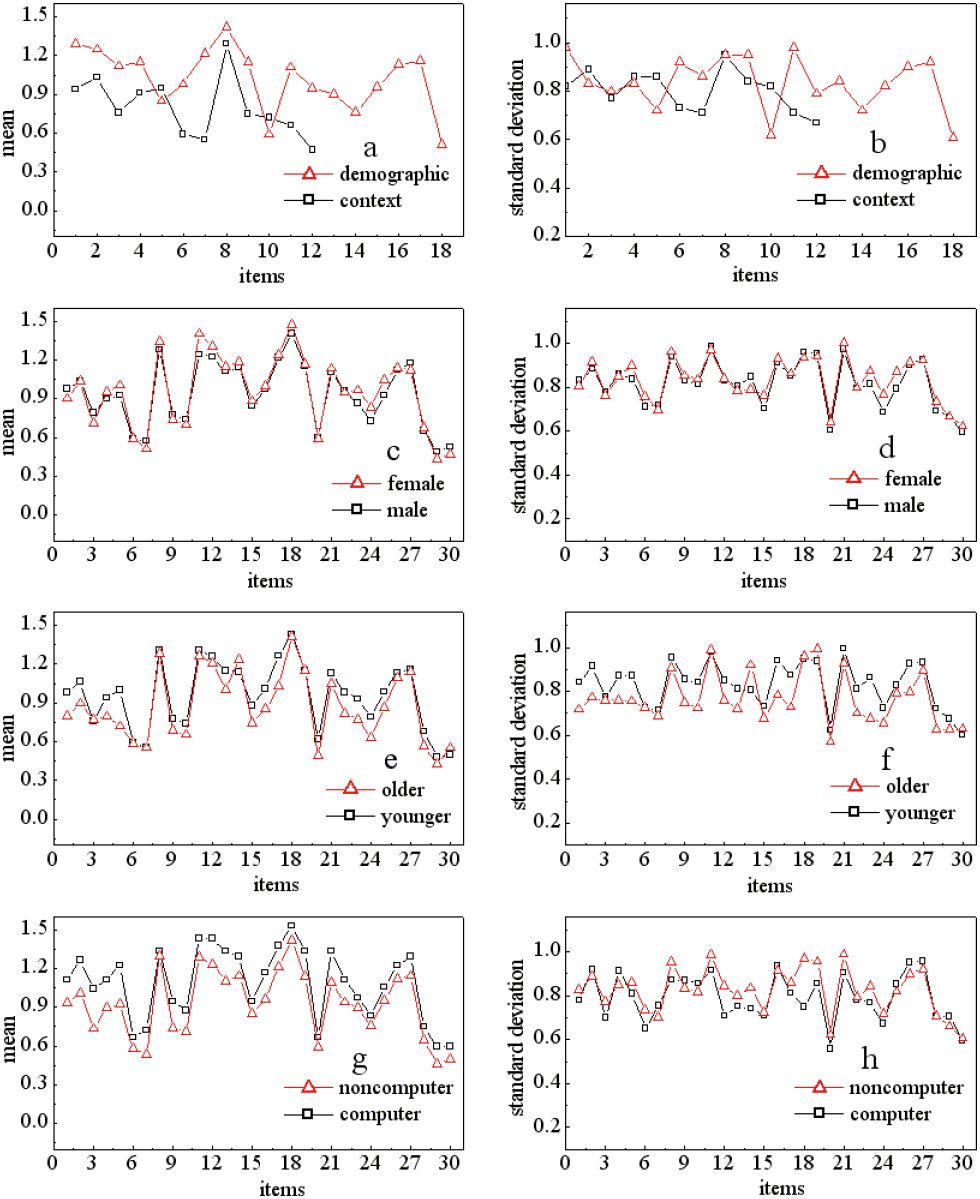
Mean and standard deviation of compared pairs with different features. *a* and *b* represent the compared values between CI and DI, *c* and *d* represent the compared values between male participants and female participants, *e* and *f* represent the compared values between older participants and younger participants, and *g* and *h* represent the compared values between computer major participants and non-computer major participants.

Will participants make different sharing decisions depending on the type of request or their own features? We calculated the mean disclosures and the values of standard deviation by dividing the data into four conditions: whether the requested item is context- or demographic-related, whether the participants are males or females, whether the participants are younger or older, and whether the participants are computer majors or non-computer majors. Fig 6 shows the comparisons of mean and standard deviation for each request under different conditions.

As the hypotheses mentioned before, we will look at the results in 4 respects:

1. Will users demonstrate different sharing behavior towards different types of items—e.g., demographic (red squares) vs. context (black squares)? The comparison is shown in Fig 6a (mean) and 6b (standard deviation), and the mean disclosures indicated that participants’ sharing volume has no relationship with the type of requested item but is strongly correlated with the sensitiveness of the requested item. In other words, the more sensitive the item is, the more difficult to collect the information from the participants. The values of standard deviation revealed that participants’ sharing behavior towards CI are more stable than the behavior towards demographics. Specifically, when the requested DI is mild or sensitive, they showed more varied behavior and less agreement on sharing decisions. As a result, we shall say that hypotheses H1a and H1b are not supported, and only the sensitiveness will affect the variability of users’ sharing behavior. Cronbach’s alpha in each type of items is 0.81 for sensitive DI, 0.81 for mild DI, 0.79 for non-sensitive DI, 0.83 for sensitive CI, 0.80 for mild CI, and 0.78 for non-sensitive CI.
2. Will male (black squares) and female (red squares) users demonstrate different sharing behavior towards the items? The mean in Fig 6c and standard deviation in Fig 6d confirm that there is no difference between males and females and show similar sharing behavior regardless of the type of information requested. As a result, hypotheses H2a and H2b, where females tend to be more conservative in the sharing behavior than males, are not supported.
3. Will younger participants (black squares) and older participants (red squares) demonstrate different sharing behavior towards the items? The answer is yes, which is verified by the mean disclosures in Fig 6e and standard deviations in Fig 6f. The younger participants tend to share much more information than the older participants, as indicated by looking at the mean values of the items, and the younger participants’ sharing behavior is more varied. As a result, hypotheses H3a and H3b are supported.
4. Will participants who majored in computer science (black squares) demonstrate different sharing behavior with the participants who did not major in computer science (red squares)? This answer also supports proposed hypotheses H4a and H4b. Participants majoring in computer science tend to share much more information than other participants. However, their behavior is less varied than that of other majors. We guess that this is because participants who major in computer science should know the consequences of sharing information and believe that they will receive generally more benefits than risks, whereas other majors know less, so they exhibit more conservative behavior.

These are good findings, because we have confirmed that users’ high disclosure and low variability could lead to high prediction accuracy in system performance. Given the difference in predicting users’ sharing partners based on age and computer/non-computer major, we could argue that nourishing the background knowledge of participants or setting up an agent to provide decision support would direct participants in a website owner’s preferred direction—e.g., requesting more information from participants without lowering their satisfaction or raising privacy concerns. Here, we will run our ISBP model to predict their potential partners.

### Hypothesis test for prediction accuracy under ISBP model

**Fig 7:**
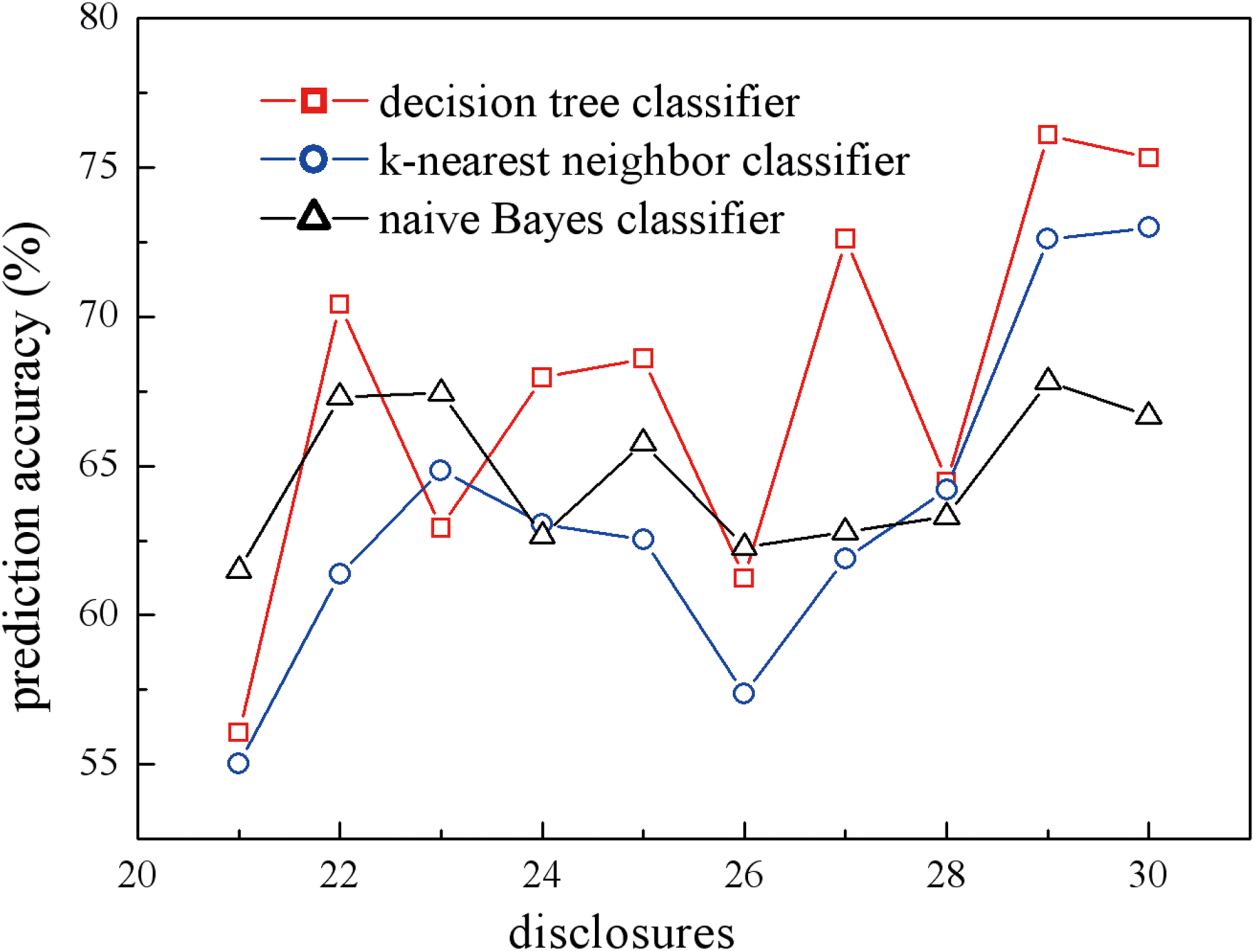
Predicting accuracy for participants’ sharing decisions. The models were built with three ISBP based ML techniques: decision tree classifier (squares), *k*-nearest neighbor (circles), and Naive Bayes classifier (stars).

We use WEKA to implement the ISBP-based ML techniques. WEKA [33] is a popular suite of ML software written in Java, with a workbench that contains a collection of visualization tool algorithms for data analysis and predictive modeling. It supports several standard data mining tasks, including the classification in this paper, and facilitates easy variation of parameters as wished to perform ISBP modeling. The formula in which we arrange the training set and the testing set for predicting a participant’s No. *X* decision is

train(gender,  age,  major,  dec 1,  dec 2, …, dec X − 1)  →  test (dec X) (5)

where *dec X* stands for participants’ sharing decision towards the *X*th requested item. All ML algorithms were run with tenfold cross-validation. The predicted decision will be sent to the participants for confirmation, and the accuracy will be calculated as the percentage of participants who acknowledge the predicted decision. To avoid the cold start problem and for warm-up purposes, we calculate the prediction accuracy for only *dec 21* to *dec 30*, and Fig 7 shows the accuracy of the ML techniques.

**Fig 8:**
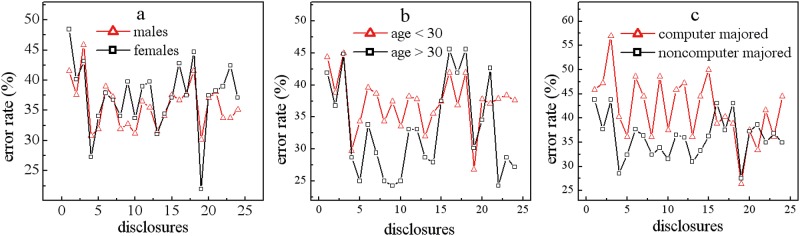
Error rates are compared in each condition. Males (triangles) vs. females (squares), younger participants (triangles) vs. older participants (squares), computer majors (triangles) vs. non-computer majors (squares).

We further pick up the errors and split them by 3 conditions—gender, age, and major—and the benefit is that the prediction accuracy is not algorithm dependent, as shown in Fig 8.

Is the prediction accuracy for sharing behavior different between males and females? In Fig 8a, the triangles represent the prediction accuracy for items 21–30 answered by male participants, and the squares represent the answers from the female participants. In most items, the values of prediction accuracy between males and females are similar, and the maximum value differences are less than 0.5%. Together with the phenomenon found in Fig 6c and 6d, in which males demonstrate a similar level of disclosure and similar variability to females, we say that users’ sharing variability is closely related to the prediction accuracy. One possible reason could be that users’ privacy calculus is closely related to their social experiences rather than the gender gap, and females and males obtain similar knowledge and social interactions in social networks.

Will the older participants’ behavior be easier to predict than that of the younger participants? This is strongly supported by Fig 8b. The prediction accuracy for more than 8 items has confirmed that the younger participants’ sharing behavior is very difficult to predict. As we mentioned before, younger participants are likely to share more information and less stable responses, and we argue that they have more difficulty in managing sharing decisions than the older participants. The differences in prediction accuracy between the younger participants and the older participants could be more than 10%. This fact also supports that the variability of users’ answers are closely correlated with the prediction accuracy. Taking one step beyond the prediction accuracy, we argue that if an agent is developed to help users’ sharing decision making in social networks, we should mainly focus on the decision support aspect for the younger participants and suggest that website owners be more careful in managing the accounts of younger customers.

Will the prediction accuracy be high when the participants are computer majors? Fig 8c supports this hypothesis by revealing a very interesting fact that participants who are computer majors are harder to predict than the non-computer major participants. This indicates that sharing knowledge could be gained by users during their answering process and directed in a fashion preferred by the website owners: disclosing more information. One trigger for this outcome may be when the system successfully skips annoying requests and maintains high satisfaction or users’ knowledge on information sharing is gained when answering our requests. If either is true, we may argue that users’ answer pattern can be nudged in a system-preferred way, so that we could further improve the agent to provide justifications to “persuade” users to give more information, and more information will help create more accurate prediction, thus developing a mutual-benefit loop.

To summarize, our ISBP model has revealed an interesting rule of users’ sharing behavior: Highly sensitive requests will cause users’ disclosures to be more varied, which further lowers the prediction accuracy of their partners, especially among younger users and non-computer majors. We will further test this argument in our prototype of the multiple-domain recommender system. This system collects users’ information for generating their trust partners, and users can brainstorm to discuss academic questions. We invited 377 people from our college campus (143 males/234 females, 216 students/161 faculty members, 109 computer majors/268 non-computer majors), and they were informed that the system will collect their information for partnership-establishing purposes and that the more information shared will guarantee a more trustworthy partner. Thirty items were requested, including 10 MI, 10 NI, and 10 SI from Table 1, and the volunteers were randomly assigned into 2 different conditions of sharing order (number of volunteers in each condition are almost identical):

Condition 1: 10 SI 91 → 10 MI → 10 NI, in which the sensitiveness of requested items are decreasing

Condition 2: 10 NI → 10 MI → 10 SI, in which the sensitiveness of requested items are increasing

**Fig 9:**
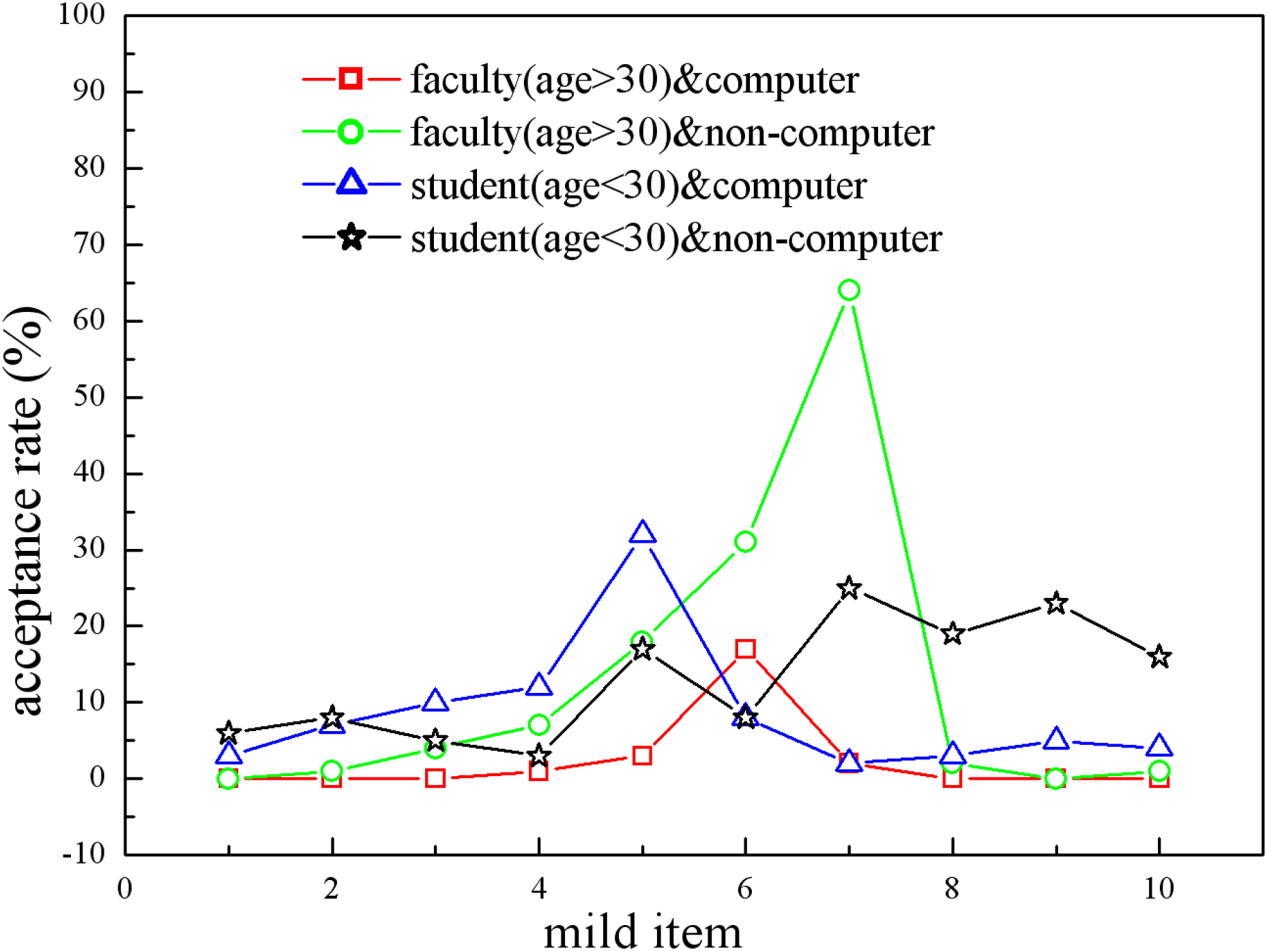
The disclosure of users in Condition 1 and Condition 2. Faculty members (older users) tend to behave more stably than students (younger users) in sharing the information, whereas computer majors are less varied than non-computer majors in information sharing. As a result, computer major faculty members’ behavior is most stable, and non-computer major students’ sharing behavior is the least stable.

All requests require the volunteer to disclose real information when he/she agrees to share. After the 15 requests, the volunteer would be shown a partner candidate from among the other users, including a brief resume, and he/she would choose to accept, and thus obtain detailed information, or deny. If one volunteer accepted the predicted partner, all disclosed information would be mutually available. We checked the IP addresses and MAC addresses to ensure that the volunteers did not attend our experiment repeatedly, and the cheating test was also applied for quality purpose. Because there was no significant difference among users towards SI and NI (the responses are almost all no or all yes), we mainly look at users’ sharing actions towards the mild items and the acceptance rate of partner candidates, as shown in Fig 9.

The results support the argument generated from the ISBP model: there is no difference between the sharing behavior of male users and female users, but the gaps between users of different ages or majors are obvious. As shown in Fig 9a, the users aged over 30 who are computer majors behave much more stably (sd = 3.17%) regarding the mild items than other users. In contrast, non-computer major students showed more varied sharing behavior towards the mild items (sd = 17.52%). To test the connection between the variability of users’ disclosure and the prediction accuracy of partners, we calculate the prediction accuracy shown in Table 2, where C/F represents computer major faculty members, C/S represents computer major students, NC/F represents non-computer major faculty, and NC/S represents non-computer major students.

**Table 2:** Prediction accuracy for users in condition 1 and condition 2.

	C/F	C/S	NC/F	NC/S
**C1**	89.37%	53.81%	65.59%	47.29%
**C2**	87.20%	60.07%	79.33%	73.85%

In condition C1, the sensitiveness of the requested items is decreasing, which caused the younger users and non-computer majors to be more conservative to the MI than in condition C2, in which the sensitiveness of the requested items is increasing. However, the older users and computer majors made similar decisions in conditions C1 and C2. As a result, we infer that the sensitive requests caused the sharing behavior of younger users and non-computer majors to be more varied but had no effect on the older users or computer majors, probably because they have knowledge that could support their decision making on disclosures. Table 2 supports this argument by showing that the values of prediction accuracy for computer major faculty members are very high under both conditions, and the varied sharing behavior reduced the prediction accuracy for partners of younger or non-computer major users. The reason why younger users and non-computer majors shared less information after seeing the sensitive items than before seeing the sensitive items is likely that they simply felt offended, so they just denied all requests without evaluating the risk and benefit, causing the system to not understand the underlying rule of sharing behavior and further reducing the prediction accuracy of their partners.

## Conclusions

This paper provides new insight into users’ privacy decisions on social networks, and we propose a model, named the information sharing behavior prediction model, that emphasizes users’ trust partnership formation and addresses the topic of predicting users’ information sharing behavior by exploring various factors—e.g., gender, age, and major. We test our hypotheses using data from two real-life datasets, and the results provide some evidence that argues not only that the amount of personal information shared is dependent on their own features but also that the predictability of users’ sharing behavior is individual-dependent, e.g., the predictability of females’ sharing behavior is similar to the predictability of males’ behavior, younger participants’ sharing behavior is more difficult to predict than older participants’ behavior, and the sharing patterns of participants who are non-computer majors are more difficult to capture than the behavior of participants who are computer majors. This study is a pioneering work that applies ML to the dataset with information sharing behavior and a guideline for applying ML techniques in WEKA, and it also could benefit researchers and faculty who concentrate on user-centered strategy analyses and human–computer interactions in information sharing studies. As a result, we recommend that researchers and website owners push forward and implement more beneficial and useful policies for information-requesting strategies and less risky voluntary options if they want to know their users better. In the era of Big Data, users tend to have registered accounts on multiple social networks, and collecting users’ data from multiple social networks will help us know them much better, which will further increase the prediction accuracy of users’ partners. The conventional tools for judging system performance would be no longer useful because the items in each social network are mostly different (people in Facebook, photos in Flickr, movies in IMDB, etc.) A proper way to test the performance of multiple domains would be to determine the amount of users’ shared information and the predictability of their sharing behavior as we do in this paper. In future work, we could establish more complicated experiments that combine users’ characteristics and attitudes to further exploit the connections between users’ lifestyles and their privacy disclosure preferences, and hopefully more interesting issues could be found regarding users’ privacy-related sharing behavior.

## Supporting Information

S1 File Dataset of 774 qualified participants. We hired 860 participants from Sojump to attend our survey, but 86 participants were eliminated from further analysis for not passing the cheating test.
(XLS)
